# iTRAQ protein profile analysis of sugar beet under salt stress: different coping mechanisms in leaves and roots

**DOI:** 10.1186/s12870-020-02552-8

**Published:** 2020-07-22

**Authors:** Junliang Li, Jie Cui, Dayou Cheng, Cuihong Dai, Tianjiao Liu, Congyu Wang, Chengfei Luo

**Affiliations:** School of Chemistry and Chemical Engineering, Harbin Institute of Technology, Harbin, 150001 China

**Keywords:** iTRAQ, Salt stress, *Beta vulgaris*, Proteomics, Differentially abundant protein species

## Abstract

**Background:**

Salinity is one of the most serious threats to world agriculture. An important sugar-yielding crop sugar beet, which shows some tolerance to salt via a mechanism that is poorly understood. Proteomics data can provide important clues that can contribute to finally understand this mechanism.

**Results:**

Differentially abundant proteins (DAPs) in sugar beet under salt stress treatment were identified in leaves (70 DAPs) and roots (76 DAPs). Functions of these DAPs were predicted, and included metabolism and cellular, environmental information and genetic information processing. We hypothesize that these processes work in concert to maintain cellular homeostasis. Some DAPs are closely related to salt resistance, such as choline monooxygenase, betaine aldehyde dehydrogenase, glutathione S-transferase (GST) and F-type H^+^-transporting ATPase. The expression pattern of ten DAPs encoding genes was consistent with the iTRAQ data.

**Conclusions:**

During sugar beet adaptation to salt stress, leaves and roots cope using distinct mechanisms of molecular metabolism regulation. This study provides significant insights into the molecular mechanism underlying the response of higher plants to salt stress, and identified some candidate proteins involved in salt stress countermeasures.

## Background

High soil salinity is one of the most severe abiotic threats to plants, reducing both yield and quality of crops [1–3]. Unlike other plant abiotic stresses, salinity causes both osmotic stress and ion toxicity via Na^+^ and Clˉ accumulation [4]. The latter may cause membrane disorganization, generation of reactive oxygen species (ROS), metabolic toxicity, inhibition of photosynthesis and attenuation of nutrient acquisition [5, 6]. However, some salt tolerant varieties such as sugar beet (*Beta vulgaris ssp. vulgaris*) can adapt to these conditions and produce a good harvest. Sugar beet is one of the most important sugar-yielding crops. As a recently domesticated crop, cultivated beets have inherited salt tolerance traits from their wild ancestor *Beta vulgaris ssp. maritima* (*B. maritima* or ‘sea beet’) [7].

Proteome analysis provides a more direct insight into molecular phenotype than transcriptome analysis. Isobaric tags for relative and absolute quantitation (iTRAQ) [8–10] is one of the most reliable labeling techniques for proteome quantification, and has been used previously to analyze salt stress-induced proteome changes in sugar beet. However, Li et al. only analyzed changes of membrane proteins in sugar beet monosomic addition line M14 [11]. In Yu et al. [12] the changes were only induced by short-term salt stress (30 min and 1 h) and examined only proteome/phosphoproteome changes in M14 leaves. Finally, Wu et al. studied proteome changes in seedlings, but only in shoots and roots, and after a very long exposure (50 mm NaCl for 72 h) [13]. While these studies are useful, we note that plant proteome responses to salt stress depend on its intensity, duration and the organ examined [14, 15]. In particular, salt treatment can be gradual (salt stress) or immediate (shock) [16]. These elicit different plant responses and may affect interpretation of results [7].

In the present study, we have used iTRAQ-based quantitative proteomic analysis to identify differentially abundant protein species (DAPs) of cultivar sugar beet ‘O68’ exposed to salt stress caused by NaCl in both leaves and roots. A total of 70 and 76 DAPs were identified in leaves and roots, respectively. The function of these DAPs were predicted using Gene Ontology (GO), Kyoto Encyclopedia of Genes and Genomes (KEGG), Clusters of Orthologous Groups of proteins (COG) and STRING. These results provide insights into the underlying molecular mechanisms of stress responses and specifically improve our understanding of the salt stress response in sugar beet.

## Results

### Effects of salinity on sugar beet physiological indexes

**Fig. 1:**
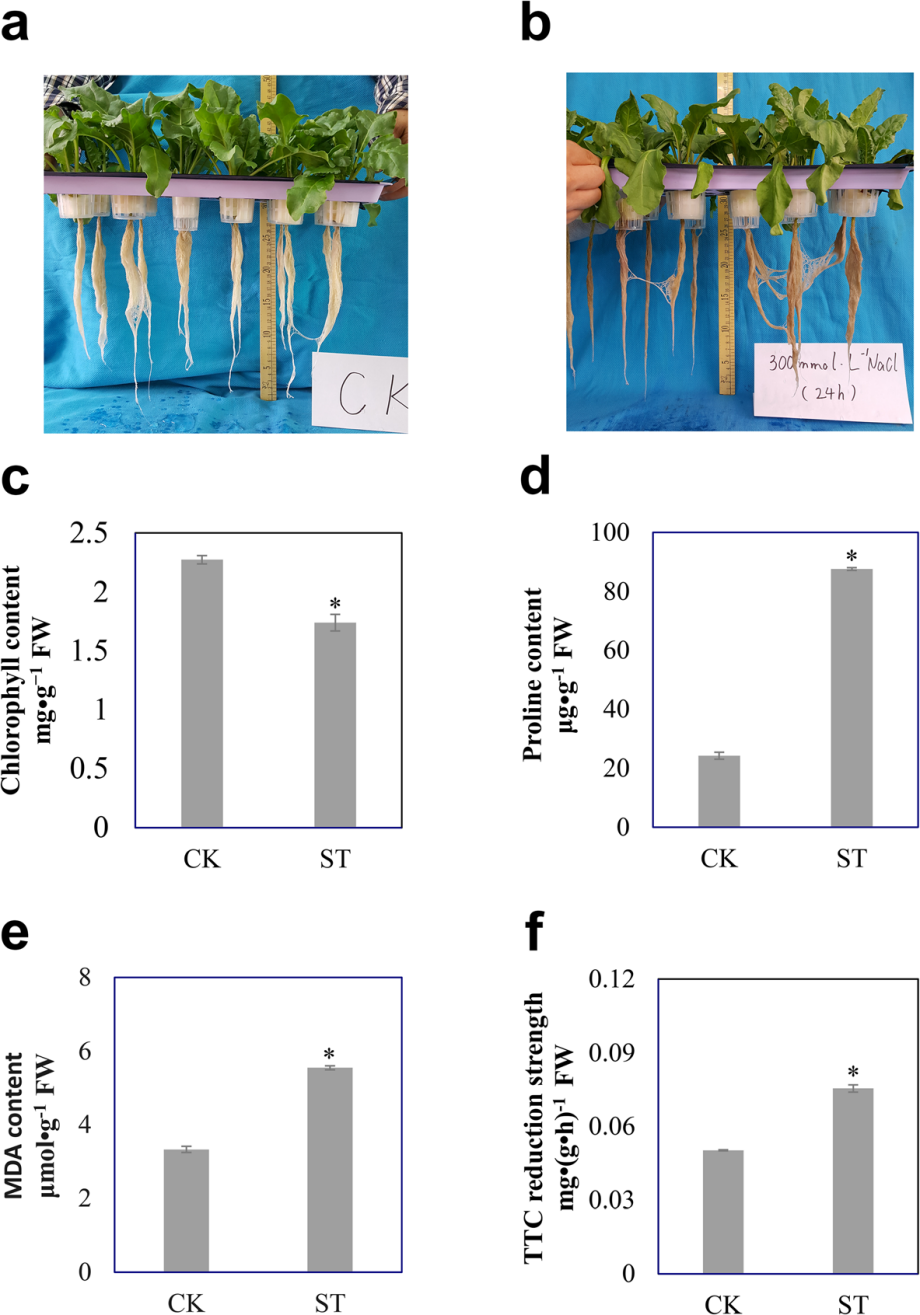
Physiological indices for sugar beet subjected to salt stress. **a** Control plants; **b** stressed plants treated with 300 mM NaCl; **c** chlorophyll; **d** malondialdehyde; **e** proline; **f** root activity. The mean and SD were calculated from three repeats of each group and the bars indicate one standard deviation. The asterisk indicates significant difference between control (CK) and salt-stressed (ST), (*p < 0.05)

Exposure of sugar beet seedlings to NaCl resulted in morphological and physiological changes in both leaves and roots, such as clear wilting of leaves and color darkening of the roots (Fig. 1a, b). In leaves, chlorophyll content was reduced to 76% compared to control plants (Fig. 1c) but displayed a 3.6-fold increase in proline (Fig. 1d) and a 1.6-fold higher content in MDA (malondialdehyde) (Fig. 1e). Activity of the roots was measured by monitoring TTC (triphenyltetrazolium chloride); reducing capacity was 1.5-fold greater under salt stress compared to control plants (Fig. 1f).

### Primary data analysis and protein identification information by iTRAQ

A total of 31,438 and 39,522 MS/MS counts were generated from leaves and roots, respectively. In leaves, 10,121 unique peptides and 3175 proteins were identified against the UniProt database, where 1966 of the proteins (61.6%) had at least two unique peptides. In roots, 13,248 unique peptides and 3935 proteins were identified, of which 2541 (64.6%) of the proteins had at least two unique peptides. The length and number distribution of the peptides is shown in Fig. S1. Statistical analyses showed that most peptides having 8–15 amino acids. The peptide number distribution of proteins indicated that 90% of identified proteins contained less than 8 segments (Fig. S2). In leaves and roots, 60 and 70 low molecular weight proteins (Mr < 10 kDa), and 300 and 417 high molecular weight proteins (Mr > 100 kDa) were identified, respectively (Fig. S3). Coverage with less than 10%, 10–30%, and 30–100% accounted for 57.6, 33.9 and 8.4% in leaves (Fig. S4a) and 55.6, 30.6 and 13.8% in roots (Fig. S4b). The distribution of protein coverage showed that coverage with less than 10, 10 -30%, and 30 100% accounted for 57.6, 33.9 and 8.4% in leaf (Fig. S4a), and 55.6, 30.6 and 13.8% in root (Fig. S4b).

### Identification of differential abundance protein species (DAPS)

**Fig. 2:**
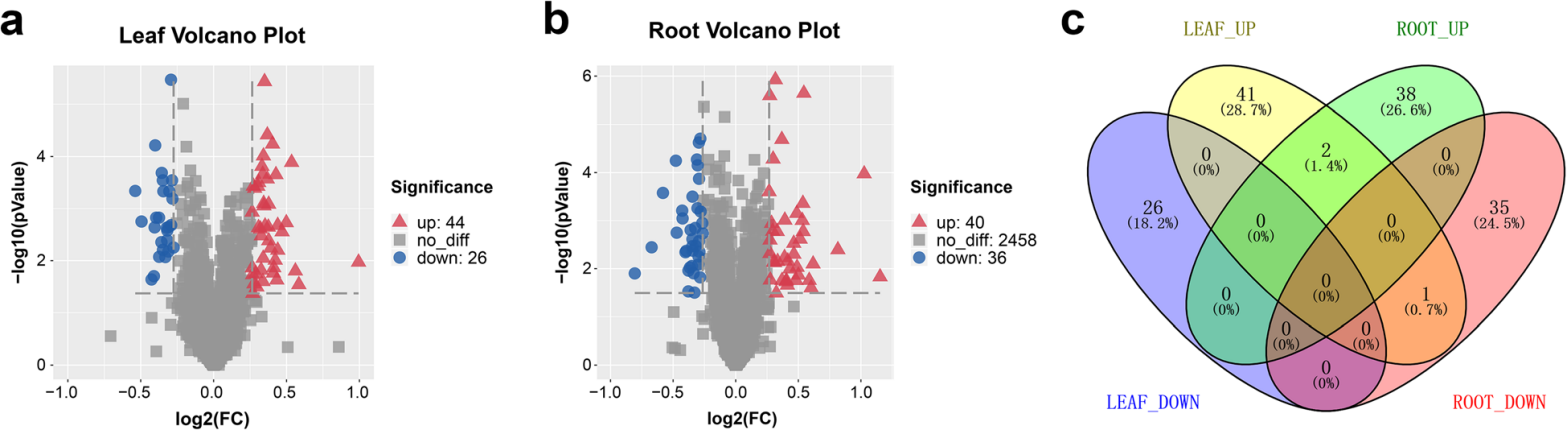
Distribution of salt-stress-responsive differentially abundant proteins (DAPs). Expression pattern of DAPs in the leaf group (**a**) and in the root group (**b**) when exposed to 300 mM NaCl; Each point represents the difference in expression between the two groups plotted against statistical significance. Proteins labeled red (up) and blue (down) show significantly different expression; **c** Venn diagram representing DAPs overlap between leaves and roots samples. Each group contains three biological replicates

Proteins with at least two unique peptides were used to screen for DAPs, using fold change > 1.2 and *p* value < 0.05. In leaves, 70 DAPs were identified, including 44 up-accumulated and 26 down-regulated (Fig. 2a). In roots, 76 DAPS were identified, of which 40 were up-regulated and 36 were down-regulated (Fig. 2b). The overlap between these DAPs is shown in Fig. 2c. For individual proteins, response to salt stress depended on their location, in leaves or in root tissue. Only two (1.4%) up-regulated proteins were found in both leaves and roots, whereas one protein (0.7%) showed opposite expression pattern. A detailed description of DAPs is shown in Tables S1 and S2.

### Bioinformatics analysis of DAPS identified by iTRAQ

**Fig. 3:**
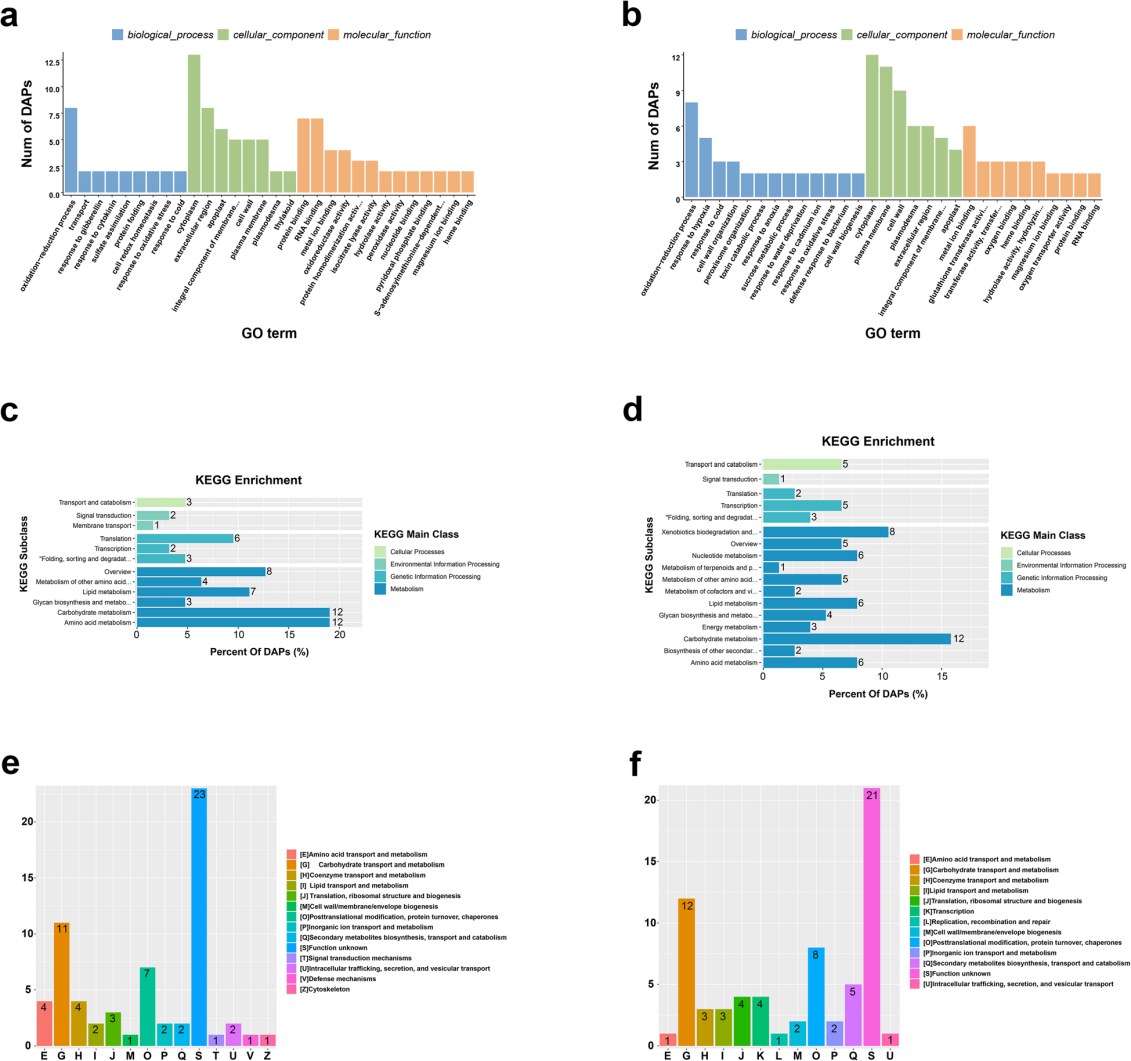
Bioinformatics Analysis of DAPS. Gene ontology (GO) enrichment of DAPs in leaves samples (**a**) and roots samples (**b**) of sugar beet. KEGG enrichment of DAPs in leaves samples (**c**) and roots samples (**d**). COG Functional Category of DAPs in leaves samples (**e**) and roots samples (**f**)

The putative functions of salt stress-responsive DAPs were investigated using GO enrichment, KEGG pathway enrichment and COG analyses (Fig. 3). GO enrichment analysis showed that oxidation-reduction process (GO:0055114) was the most significantly enriched term under the biological processes in both leaves and roots. In addition, transport (GO:0006810), response to gibberellin (GO:0009739), response to cytokinins (GO:0009735), sulfate assimilation (GO:0000103) and cell redox homeostasis (GO:0045454) were enriched in leaves (Fig. 3a). Response to hypoxia (GO:0001666), cell wall organization (GO:0071555), peroxisome organization (GO:0007031), toxin catabolic process (GO:0009407), response to anoxia (GO:0034059), sucrose metabolic process (GO:0005985), response to water deprivation (GO:0009414) and defense response to bacteria (GO:0042742) were enriched in roots (Fig. 3b). Leaves and roots had similar enrichment in cytoplasm (GO:0005737), plasma membrane (GO:0005886), cell wall (GO:0005618), plasmodesma (GO:0009506), extracellular region (GO:0005576), integral component of membrane (GO:0016021), and apoplast (GO:0048046), but thylakoid (GO:0009579) was enriched only in leaves. The most enriched molecular function categories in leaves were protein binding (GO:0005515) and RNA binding (GO:0003723). In roots, these were metal ion binding (GO:0046872) and glutathione transferase activity (GO:0004364).

Using KEGG, DAPs in both leaves and roots were divided into four main categories: Cellular Processes, Environmental Information Processing, Genetic Information Processing, and Metabolism. Specifically, 70 DAPS in leaves were further divided into 12 subclasses and mapped to 43 pathways (Fig. 3c), whereas 76 DAPs in roots were further divided into 17 subclasses and mapped to 45 pathways (Fig. 3d). A pathway related to membrane transport (ko02010) was specially enriched in leaves, and there were more metabolism-related pathways enriched in roots.

The 70 and 76 DAPS found in leaves and roots were classified into 14 and 13 COG categories, respectively. Among these, the largest group in both locations were Function unknown, Carbohydrate transport and metabolism, Posttranslational modification, protein turnover and chaperones. However, leaves contained DAPs classified into Defense mechanisms and Cytoskeleton (Fig. 3e) whereas roots contained DAPs classified into Transcription (Fig. 3f).

**Fig. 4:**
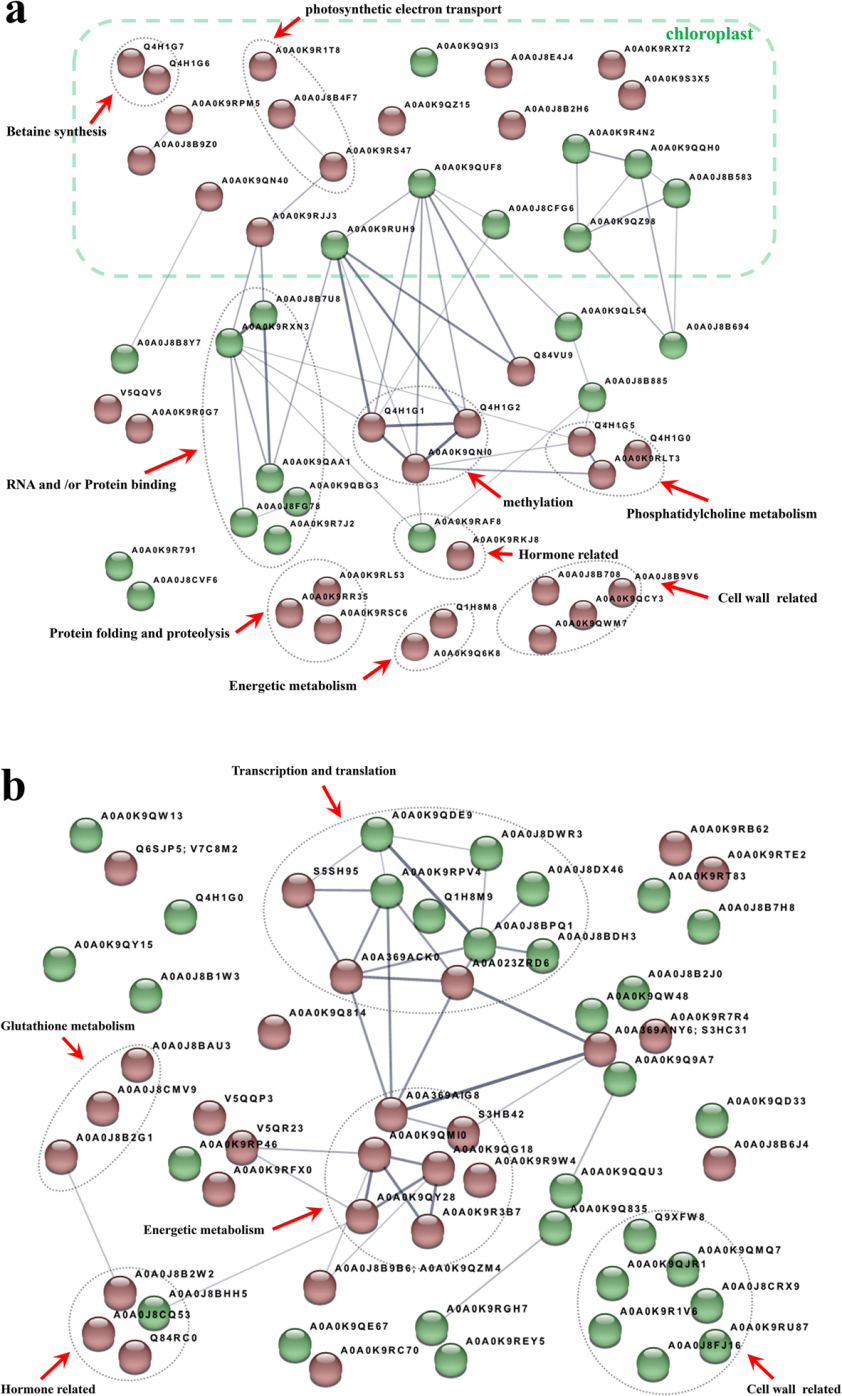
Protein–protein interaction (PPI) analysis of salt stress response DAPs in leaves (**a**) and roots (**b**). The circles represent proteins with red representing up-regulated and green representing down-regulated. The thickness of line indicates the strength of data support

The STRING protein interaction database was used to analyse protein-protein interactions (PPI) [17] for all DAPs in leaves and roots, where 31 and 26 interacting proteins were identified, respectively (Fig. 4). Among these DAPs, eight KEGG pathways were significant enriched in leaves including: Biosynthesis of amino acids (bvg01230), Metabolic pathways (bvg01100), Cysteine and methionine metabolism (bvg00270), Biosynthesis of secondary metabolites (bvg01110), Carbon metabolism (bvg01200), Glycerophospholipid metabolism (bvg00564), Sulfur metabolism (bvg00920) and Photosynthesis (bvg00195). Six KEGG pathways were significantly enriched in roots including: Glycolysis / Gluconeogenesis (bvg00010), Metabolic pathways (bvg01100), Purine metabolism (bvg00230), Biosynthesis of secondary metabolites (bvg01110), RNA polymerase (bvg03020) and Pyrimidine metabolism (bvg00240).

### Transcriptional analyses of the corresponding genes encoding DAPS

To correlate DAPs abundance and transcript levels of their corresponding genes, twelve DAPs (six each from leaves and roots, where one was common to both) were selected for qRT-PCR analysis. The same expression trend between transcript and protein levels was observed for ten of the twelve selected DAPs, whereas the other two DAPs showed no significant changes at the transcript level (Table 1). This discrepancy may be due to temporal differences to the salt stress response between transcription and translation.

**Table 1:** Comparison of expression pattern at the mRNA and protein level of DAPS

Majority protein IDs	Flod Change (ST/CK)	pval	qPCR 2-ΔΔCt	pval	trends	
					iTRAQ	qPCR
LEAF						
tr|A0A0J8B583|A0A0J8B583_BETVU	0.81	0.000	0.51	0.017	–	–
tr|A0A0J8CVF6|A0A0J8CVF6_BETVU	0.82	0.002	0.31	0.001	–	–
tr|A0A0J8B8Y7|A0A0J8B8Y7_BETVU	0.79	0.000	0.96	0.388	–	=
tr|Q4H1G6|Q4H1G6_BETVU	1.48	0.016	15.84	0.000	+	+
tr|Q4H1G0|Q4H1G0_BETVU	1.45	0.000	10.47	0.001	+	+
tr|A0A0J8B9V6|A0A0J8B9V6_BETVU	1.22	0.000	3.02	0.001	+	+
ROOT						
tr|A0A0J8B2J0|A0A0J8B2J0_BETVU	0.81	0.000	0.97	0.522	–	=
tr|A0A0J8BHH5|A0A0J8BHH5_BETVU	0.80	0.001	0.22	0.000	–	–
tr|A0A0J8CRX9|A0A0J8CRX9_BETVU	0.79	0.004	0.32	0.002	–	–
tr|A0A0J8BAU3|A0A0J8BAU3_BETVU	1.21	0.003	3.20	0.004	+	+
tr|Q4H1G6|Q4H1G6_BETVU	1.21	0.005	9.62	0.001	+	+
tr|Q84RC0|Q84RC0_BETVU	1.50	0.017	7.07	0.001	+	+

*-: down-regulated, =: no significantly change, +: up-regulated

## Discussion

Salt stress may have distinct effects on different organs of plants. Herein, we have identified 1966 and 2541 proteins in leaves and roots that possessed more than two unique peptides. Furthermore, 70 and 76 DAPs were identified in leaves and roots, respectively, of which only three were differentially expressed in both of them. Functional divergence of the proteins in leaves and roots suggested distinct responses to salt stress and different contributions to stress-resistance in sugar beet. While we are aware that owing to the technical limitations of iTRAQ these results may not represent the entire landscape of protein patterns in leaves and roots under salt stress adaptation, the possible biological significance of some key DAPS and their relevant metabolic pathways are discussed below.

### Analysis of the DAPs response to salt stress in chloroplast

Chloroplasts only exist in leaves and are the most sensitive organelles to salt stress in plants [18]. We have shown that chlorophyll content in leaves decreased 0.76-fold under high salt stress, consistent with the fact that high salinity destroys chloroplasts and affects photosynthesis [19]. Unsurprisingly, 22 of the identified DAPs in leaves were related to chloroplasts, 14 of which were up-regulated and 8 down-regulated. Three proteins, psbQ-like protein 1 (A0A0K9RS47), Plastocyanin (A0A0J8B4F7) and NAD(P)H quinone oxidoreductase subunit U (A0A0K9R1T8) from the photosynthetic electron transport chain were up-regulated under salt-stress, which may represent efforts to maintain photosynthesis. In addition, the STRING network analysis showed that psbQ-like protein 1 interacted with another up-regulated protein, peptidyl-prolyl cis-trans isomerase fkbp16–4 (PPI) (A0A0K9RJJ) (Fig. 4a), consistent with previous studies [20]. This suggests that plants respond to salt stress by increasing PPIs in order to accelerate protein synthesis. Another up-regulated protein is thioredoxin Y1 (A0A0K9QN40), which regulates the activity of photosynthetic enzymes [21]. Furthermore, DNA repair RAD52-like protein (A0A0K9RXT2) and DNA-damage-repair/toleration protein DRT100-like (A0A0K9S3X5) were also up-regulated proteins in the chloroplast. These DAPs may help protect the chloroplast DNA from damage under salt stress and enhance salinity tolerance [22, 23].

Glycine betaine, considered to be the best osmotic regulator, is not only involved in osmotic regulation of cells but also in the stabilization of macromolecules. For example, it protects the major enzymes and terminal oxidases of the TCA (tricarboxylic acid) cycle and stabilizes the peripheral peptides of the light system [24–26]. In plants, betaine is produced from choline via two oxidation steps and catalyzed by two enzymes that are significantly up-regulated under salt-stress: choline monooxygenase (Q4H1G6) and betaine aldehyde dehydrogenase (Q4H1G7) [27–29]. In addition, SEX4 (STARCH-EXCESS 4, also known as Dual specificity protein phosphatase 4, DSP4) (A0A0J8B9Z0) acts as a bridge between light-induced redox changes and protein phosphorylation in the regulation of starch accumulation [30]. SEX4 may help promote the decomposition of transitory starch into soluble sugar, in order to regulate osmotic pressure in plant cells.

Another up-regulated protein, LS (6,7-dimethyl-8-ribityllumazine synthase) (A0A0J8E4J4), has been shown to catalyze the penultimate step in the synthesis of riboflavin and regulates intracellular REDOX reactions. In addition, LS plays a role in the JA signaling pathway and participates in plant defense reactions [31]. The ABC transporter B family member 26 (A0A0K9QZ15) was also up-regulated, which may be related to specific transport functions. THI1 (Thiamine thiazole synthase) (A0A0K9Q9I3) was down-regulated. This protein takes part in both guard cell abscisic acid (ABA) signaling and drought response in *Arabidopsis* [32]. Finally, enolase 1 (A0A0J8CFG6) in plastids was down-regulated, consistent with previous reports [33, 34].

### Analysis of salt stress resistant DAPs

Salt stress affects the normal development of plants in the form of osmotic imbalances, ion injury and reactive oxygen species (ROS) formation. Soluble sugar and proline, like betaine, are also essential osmotic regulators. A 3.6-fold increase in proline was detected in leaves, although proline metabolism-related enzymes (like *P5CS*) did not accumulate in either leaves or roots. We did find two differentially accumulated sucrose synthases (Q6SJP5 and V7C8M2) in roots. Sucrose synthase (SuSy) is a widely distributed glycosyltransferase in plants that catalyzes the decomposition and synthesis of sucrose. Accumulation of SuSy under abiotic stress has been found in several plants, especially in roots [35–37]. SuSy is not only involved in osmotic regulation of plants, but also functions at a branch point to allocate sucrose to either cell wall biosynthesis or glycolysis [38]. Thus, in sugar beets, choline monooxygenase and betaine aldehyde dehydrogenase may play a role in osmotic regulation of leaves, whereas SuSy may be important in the osmotic regulation of roots.

The toxicity of NaCl to plants is mainly caused by sodium and chloride ions, as well as ROS production. In leaves, the observed 1.6-fold increase in MDA reflects the oxidative damage caused by stress. In plants, excess ingestion of Na^+^ can affect the absorption of mineral nutrients such as Ca^2+^, Mg^2+^ and K^+^ [39]. However, being a salt-tolerant plant, sugar beet can use Na^+^ instead of K^+^ for osmotic regulation, stomatal regulation and long distance transport of anions [40–42]. Consistent with the fact that high levels of chloride can inhibit the uptake of NO_3_^−^, a significant decrease in high affinity nitrate transporter (A0A0J8B2J0) was observed in roots. Increase of ROS and oxidative bursts can affect photosynthesis, metabolism and signal transduction. However, plants have their own detoxification system. First, exogenous toxins or cytotoxins are metabolized by enzymes such as cytochrome P450 monooxygenase. Second, enzymes like GST catalyze coupling reactions between processed products and sugar (or GSH). Third, these conjugates are recognized by ATP coupling transporters and are transported to vacuoles or secreted [43, 44]. Two CYP family members (A0A0K9RP46 and A0A0K9RFX0), 3 GST family members (A0A0J8B2G1; A0A0J8CMV9; A0A0J8BAU3) and an F-type H^+^-transporting ATPase (A0A369ANY6) were differentially expressed. Root activity results showed a 1.5-fold increase in TTC reduction capacity, likely due to accumulation of GSTs. Thus, these proteins may play an important role in detoxification against salt stress in roots.

Sugar beets respond to salt stress by compartmentalization [45]. In general, a higher salt content is found in petioles and older leaves whereas lower salt is found in new leaves, which ensures their correct function [46]. Unlike what was found in roots, in leaves there was no accumulation of CYP and GST, but differential expressions of two peroxidase family members (A0A0K9R0G7 and A0A0J8B8Y7). Chalcone synthase (CHS) (A0A0K9R791) and flavanone-3-hydroxylase (F3H) (A0A0J8CVF6) were detected in leaves. These are key enzymes in the metabolism of flavonoids, which play an important role in non-enzymatic scavenging of ROS [47, 48], therefore these proteins may be involved in detoxification. This finding may be due to the sampling of functional leaves (the third-pair euphylla). Further studies are needed to determine the differences between new and old leaves.

Plants also use overexpression of non-symbiotic hemoglobin (NsHb) as a strategy to reduce the damage caused by oxidative stress, by improving the activity of the antioxidant enzyme system [49–51]. One non-symbiotic hemoglobin protein, V5QQV5, was up-regulated in leaves, whereas two, V5QQP3 and V5QR23, were found in roots. The latter was upregulated more than two-fold, suggesting an underestimated role in the resistance against salt stress.

### Analysis of the DAPs associated with Apoplast and cell wall

GO analysis results showed that, in both roots and leaves, a large number of DAPs associate with apoplast and cell wall. However, cell wall DAPs in root and leave responded differently to salt stress. The apoplast is the first plant compartment encountering environmental signals [52], and apoplast proteins are involved in the response to these signals and in the perception and transduction of signals together with the plasma membrane [53, 54]. Stress signals are first detected by the cell wall, which transmits them into cells to regulate their activity [55, 56]. Interestingly, DAPs related to apoplast and cell wall were up-regulated in leaves. Specifically, β-galactosidase (A0A0J8B708), β-D-xylosidase 5 (A0A0K9QCY3), endo-1,3;1,4-β-D-glucanase (A0A0J8B9V6), and xyloglucan endotransglucosylase/ hydrolase protein 24-like (A0A0K9QWM7) were significantly accumulated. In higher plants, β-galactosidase is the only enzyme that can cleave β-1,4-galactosan internally and removes galactose residues from cell wall polysaccharides [57]. Xylan is the main polysaccharide in plant cell walls, and β-D-xylosidase is an O-glycosyl hydrolases that hydrolyzes glycosyl bonds in xylans [58]. Endo-1,3(4)-β-D-glucanase has a specific digestive effect on cellulose microfibers and plays an important role in regulating plant cell wall structure [59]. Xyloglucan endotransglucosylase/hydrolases (XTHs) play an essential role in the formation of xyloglucan cross-links [60]. Up-regulation of these genes in sugar beet leaves suggests a response to salt stress that results in maintaining the ductility of cell walls. Leaf cells may increase in volume to compensate for chlorophyll damage, thus ensuring energy supply.

In contrast, down-regulation of the following DAPs suggest that sugar beet roots resist salt stress by inhibiting cell wall relaxation. Indeed, α-xylosidase 1 (A0A0K9RU87), xyloglucan endotransglucosylase/ hydrolase (A0A0J8CRX9 and A0A0K9QMQ7), β-galactosidase 5 (A0A0K9R1V6), Expansin-like A2 (A0A0K9QJR1) and proline-rich protein (PRP) 3 (A0A0J8FJ16) were down-regulated. Expansin is a cell wall relaxation protein and its accumulation is a biochemical mechanism for salt tolerance in wheat varieties [61], whereas PRP is a structural protein involved in cell wall construction and defense.

### Analysis of DAPs related to metabolism

Consistent with the fact that DAPs involved in carbohydrate and energy metabolism are indispensable, we found up-regulation of NADH-ubiquinone reductase complex 1 MLRQ subunit (Q1H8M8) and Cytochrome c oxidase subunit 5C (A0A0K9Q6K8) in leaves. In roots, up-regulation was found for 2 EMP components 6-phosphofructokinase (A0A0K9R9W4) and glyceraldehyde-3-phosphate dehydrogenase (A0A0K9R3B7), succinyl-CoA ligase, β subunit (S3HB42) belonging to TCA, ADH (A0A0K9QY28), PDC1 (A0A0K9QG18) and PDC2 (A0A0K9QMI0), ATP synthase α (A0A369AIG8) and β subunits (S3HC31). PDC (Pyruvate decarboxylase) and ADH (Alcohol dehydrogenase) can switch production of lactic acid to ethanol, which is much less toxic to plants, or to intermediate acetaldehyde. These results indicate that sugar beet adapts to salt stress by improving energy metabolism.

Phosphatidylcholine (PC) has not only a structural role in membranes, but is the source of signaling molecules. Serine decarboxylase (SDC: Q4H1G0) catalyzes the first step in PC biosynthesis: the conversion of serine into ethanolamine [62]. Choline /ethanolamine kinase (CEK: A0A0K9RLT3) catalyzes the initial reaction step of choline metabolism to produce phosphoethanolamine [63]. Phosphoethanolamine N-methyltransferase (PEAMTs: Q4H1G5) is a rate-limiting enzyme that catalyzes the production of choline from phosphoethanolamine [64]. In leaves, up-regulation of SDC, CEK and PEAMTs may be related to both cell membrane synthesis and to the synthesis of betaine and phosphatidic acid (PA). Unsurprisingly, SDC (Q4H1G0) and GPI ethanolamine phosphate transferase 1 isoform X2 (A0A0J8B1W3) were down-regulated in roots as well as dirigent protein (A0A0K9QD33), involved in yielding lignans. These results are consistent with our analysis of cell wall-related DAPs in that, under salt stress, leaf cells strive to increase volume while root cells maintain it.

Similarly, DAPs involved in protein folding and degradation were increased in leaves, e.g., Tubulin-folding cofactor D (A0A0K9RSC6), Aspartic proteinase nepenthesin-1 (A0A0K9RL53) and Ubiquitin carboxyl-terminal hydrolase 12-like (A0A0K9RR35). In contrast, prefoldin subunit 4 isoform X1 (A0A0K9RGH7), DnaJ protein homolog ANJ1(A0A0K9REY5) and Basic 7S globulin 2-like (A0A0K9QE67) were down-regulated in roots.

### Analysis of DAPs involved in transcription and translation processes

Plants require continuous adaptation to the environment, and one mechanism is by regulating transcription and translation. In leaves, we observed down-regulation of six DAPs associated with RNA and /or Protein binding, especially Glycine-rich RNA-binding protein 2 (GR-RBP2) (A0A0J8FG78), which can affect the expression of genes encoded by mitochondrial genome and thus regulate respiration [65]. GR-RBP plays a remarkable role in the response to stress [66, 67]. In roots, a number of DAPs involved in transcription and translation were down-regulated, like DNA-directed RNA polymerases II, IV and V subunit 3 (A0A0K9RPV4), DEAD-box ATP-dependent RNA helicase 7 (A0A0J8BDH3) or H/ACA ribonucleoprotein complex subunit 2-like protein (A0A0J8BPQ1). Also, unlike in leaves, 50S ribosomal protein L14 (A0A023ZRD6) and 50S ribosomal protein L22 (A0A369ACK0) were higher than in the control. Since these two ribosomes are located in the mitochondria, we hypothesize that global transcription and translation in sugar beet is reduced during salt stress, whereas root cells locally enhance the synthesis of mitochondrial-related proteins. Such specific regulation may help ensure the proper functioning of mitochondria to obtain sufficient energy.

### Analysis of DAPs in regard to plant hormones

Plant hormones are active substances induced by specific environmental signals and have obvious physiological effects at very low concentrations. Gibberellin regulated protein (A0A0K9RKJ8) was observed to accumulate in the leaves. In roots, the enzyme 1-aminocyclopropane-1-carboxylate oxidase (ACO) is involved in the ethylene biosynthesis pathway, whereas long chain acyl-CoA synthetase (LACS) can activate the biosynthetic precursors of jasmonic acid (JA) [68]. ACO1 (A0A0J8B2W2), LACS 4-like (A0A0K9RTE2) and auxin-binding protein ABP19a (Q84RC0) were up-regulated, whereas abscisic acid receptor PYL4 (A0A0J8BHH5) was down-regulated. Both leaves and roots showed increased carboxylesterase 1(A0A0J8CQ53), which can demethylate inactive methyl salicylate (MeSA) and methyl jasmonate (MeJA) into active salicylate acid and jasmonic acid.

## Conclusion

We have used iTRAQ to reveal the divergent responses to salt stress in sugar beets leaves and roots, where 70 and 76 DAPs were identified, respectively. Leaves and roots exhibit different coping strategies under salt stress. Leaves show a relatively robust metabolism at global level, particularly in ensuring photosynthesis, to obtain the energy necessary to cope with environmental pressure. The homeostasis of leaf cells may be attributed mainly to the accumulation of betaine and ROS scavenging using enzymatic and non-enzymatic systems. Roots exhibit a relatively less active metabolism at global level. The accumulation of GST family members in root may be conducive to the survival of the root. Studies on the function of DAPs found in this study will be helpful to explore the mechanisms of beet resistance to salt stress. Our analysis enhances our understanding of the molecular mechanisms in response to salt stress in different organs of sugar beet which will enable future improvement of salt tolerance.

## Methods

### Plant materials and treatments

Cultivar ‘O68’ is an excellent parent used in traditional crossbreeding with strong salt tolerance and therefore a good choice for studying the mechanism of salt stress response in sugar beet. Treated with 300 m mol·L^− 1^ NaCl, the relative germination rate of this cultivar was more than 70% and the seedling can grow normally [69]. In addition, it has a strong regeneration capability of petiole explants which is ideally suited for use in molecular breeding. The seeds, from our own laboratory (Heilongjiang, China), were soaked in water for ten hours, sterilized in 0.1% (v/v) HgCl_2_ for 10 min, washed repeatedly with distilled water and germinated on wet filter paper in a germination box at 26 °C for 2 days. After germination, budding seeds were transferred to plastic pots (45 cm × 20 cm × 14 cm, at 12 plants per pot) and filled with quarter-strength Hoagland solution. The germinating seeds were cultivated under a 16/8 light photoperiod at 24 °C (day)/18 °C (night) in a phytotron (Friocell 707, Germany). The increase of salt concentration in Nature usually occurs gradually. Therefore, to make the salt treatment closer to that found in Nature, four-week-old plants (three-pairs-euphylla) were treated with half-strength Hoagland medium supplemented with increasing concentrations of NaCl (increase of 50 mM every 12 h), until the final concentration of 300 mM was reached. After this, treatments were continued for 24 h. NaCl-free nutrient solution was used as a control. Leaves, representing the third pair of euphylla, and roots were collected, immediately frozen in liquid nitrogen and stored at − 80 °C until further use.

### Physiologic indices detection

Different sample weights of fresh leaves from the third pair of euphylla were used to detect chlorophyll content by acetone extraction (0.1 g), proline content by ninhydrin colorimetry method (0.5 g), and malondialdehyde content using thiobarbital acid method (1 g) [70]. Fresh roots (0.5 g) were collected for the determination of root activity by TTC reduction method [70]. Data was obtained by a UV-2100PC ultraviolet-visible spectrophotometer (UNICO. LTD) and each treatment was repeated three times.

### Protein extraction, protein digestion and iTRAQ labeling

ITRAQ analysis was carried out at LC Sciences (Hangzhou, China). Leaf or root tissue from every ten plants was pooled as one biological replicate. Three biological replicates were conducted for iTRAQ-based comparative proteomics analysis. The total proteins of the leaves and roots from each sample were extracted as described [71]. Protein concentration was determined using a BioDrop μLite microdetector (BioDrop, UK), and protein quality was measured with sodium dodecyl sulfate-polyacrylamide gel electrophoresis (SDS-PAGE). For digestion, 100 μg protein of each sample was reduced with 20 mM dithiothreitol (DTT) at 37 °C for 60 min and alkylated with 40 mM (final concentration) iodoacetamide at room temperature for 30 min in the dark. The protein pool of each sample was digested at 37 °C for 12 h with Sequencing Grade Modified Trypsin using a mass ratio protein: trypsin of 100:1, and digested again for 4 h. After trypsin digestion, the peptide was desalted in a Waters sep-Pak C18 column (Waters Inc., US) and vacuum-dried. The peptide was reconstituted in 40 μl 100 mM TEAB and processed according to the manufacturer’s protocol for 8-plex iTRAQ kit (AB sciex Inc., US). iTRAQ reagents 114, 115, and 116 were used to label the peptides from CK replicates (leaf and root), and iTRAQ reagents 117, 118 and 121 were used to label the peptides from salt stressed replicates (leaf and root). The labeled peptide mixtures were desalted by Waters sep-Pak C18 column and vacuum-dried.

### Nano-LC-ESI-MS/MS analysis

The powder of labeled peptide was dissolved with 52 μl of 10 mM ammonium formate, pH 10, and fractionated in a Waters E2695 liquid chromatography system using BEH C18 chromatographic column (5 μm, 4.6*250 mm, Waters Inc). Peptides were combined into 17 fractions in each leaf sample and 18 fractions in each root sample and were freeze-dried. Each fraction was redissolved with 5 μl 0.1% formic acid (FA) and passed through a NanoLC-MS/MS system using EASY-nLC 1000 coupled to Q Exactive (Thermo Scientific, US) mass spectrometer. The eluent was sprayed via ESI source at the 2.0 kV electrospray voltage and analyzed by tandem mass spectrometry (MS/MS) in Q Exactive. The MS scan spectra ranged from 350 to 1800 m/z and was acquired in the Orbitrap with a resolution of 70,000. The dd-MS2 scan spectra was automatic selected and the dd-MS2 resolution was 17,500.

### Protein identification and quantification

MaxQuant (version 1.5.5.1) was used for iTRAQ protein identification and quantification [72, 73]. For protein identification, the *B. vulgaris* protein database of UniProt was used with the criterion of false discovery rate (FDR) < 0.01. The parameters for library searching were as follows: fixed modifications include carbamidomethyl on cysteine residues, iTRAQ 8 plex (N-term) and iTRAQ 8 plex (K); oxidative modification on methionine was set as a variable modification. The peptide mass tolerance was ±10 ppm and the fragment mass tolerance was 0.2 Da.

### Bioinformatics analysis

The biological and functional properties of proteins were analyzed with GO and KEGG databases [74, 75]. A hypergeometric test was used to find significantly enriched GO terms and KEGG pathways of DAPs. If the *p*-value was < 0.05, the GO term or KEEG pathway were regarded as a significant enrichment of DAPs. Clusters of Orthologous Groups of Proteins System was also employed for the functional classification of the DAPs.

### RNA extraction and qRT-PCR

Total RNA was extracted from leaves and roots by the MiniBEST Plant RNA Extraction Kit (TaKaRa, Japan). Approximately 2 μg total RNA was reverse-transcribed using High-Capacity cDNA Reverse Transcription Kits in a 20 μl of reaction volume (ThermoFisher Scientific, US). The reactions were incubated for 10 min at 25 °C, followed by 37 °C for 120 min, and terminated at 85 °C for 5 min. All the primers are listed in Table S3. *PP2A*+ *UBQ5* and *PP2A* + *25S* were used as endogenous controls in leaves and roots, respectively. For qRT-PCR, gene-specific primers were designed using Primer-BLAST online [76]. The qRT-PCR reactions were performed using iTaq Universal SYBR® Green Supermix (BIO-RAD, Hercules, CA) on the CFX Real-time PCR system (BIO-RAD, CA). Non-specific amplification was prevented by running a melting curve for each PCR product. The expression level of the miRNAs in different samples was calculated following the comparative 2^−△△CT^ method.

### Data treatment and statistical analysis

For the data of the physiological parameters and qPCR analysis, the mean and SD were calculated from three repeats of each treatment, and the differences were analyzed by Duncan’s multiple range test (*p* < 0.05) and an independent-samples t-test (*p <* 0.05).

## Supplementary information

**Additional file 1 Fig. S1.** Distribution of peptide length in leaf (a) and root (b) of *B. vulgaris*. **Fig. S2.** Distribution of peptide number in leaf (a) and root (b) of *B. vulgaris*. Distribution of protein mass in leaf (a) and root (b) of *B. vulgaris*. **Fig. S4.** Distribution of protein coverage in leaf (a) and root (b) of *B. vulgaris*.

**Additional file 2 Table S1.** Detailed information of DPAs in leaves.

**Additional file 3 Table S2**. Detailed information of DPAs in roots.

**Additional file 4 Table S3**. List of primers used for qRT-PCR experiments.

## Data Availability

The materials used during the current study will be freely available upon request to corresponding author: cuijie2006@163.com. The mass spectrometry proteomics data have been deposited to the ProteomeXchange Consortium via the iProX partner repository [77] with the dataset identifier PXD017954 (https://www.iprox.org/page/project.html?id=IPX0002058000).

## Abbreviations

*B. vulgaris*: *Beta vulgaris ssp. vulgaris*
iTRAQ: Isobaric tag for relative and absolute quantification
DAPs: Differential abundance protein species
GO: Gene Ontology
KEGG: Kyoto Encyclopedia of Genes and Genomes
COG: Clusters of Orthologous Groups of proteins
MDA: Malondialdehyde
TTC: Triphenyltetrazolium chloride
FDR: False discovery rate
qRT-PCR: Quantitative RT-PCR
