# Supplementary material for: iTRAQ protein profile analysis of sugar beet under salt stress: different coping mechanisms in leaves and roots
Source: BMC Plant Biol. 2020 Jul 22;20:347. doi: 10.1186/s12870-020-02552-8 (PMC7376716; doi:10.1186/s12870-020-02552-8)
Supplement: Supplementary file 1 — Additional file 1 Fig. S1. Distribution of peptide length in leaf (a) and root (b) of B. vulgaris. Fig. S2. Distribution of peptide number in leaf (a) and root (b) of B. vulgaris. Distribution of protein mass in leaf (a) and root (b) of B. vulgaris. Fig. S4. Distribution of protein coverage in leaf (a) and root (b) of B. vulgaris. [file 12870_2020_2552_MOESM1_ESM.pdf]

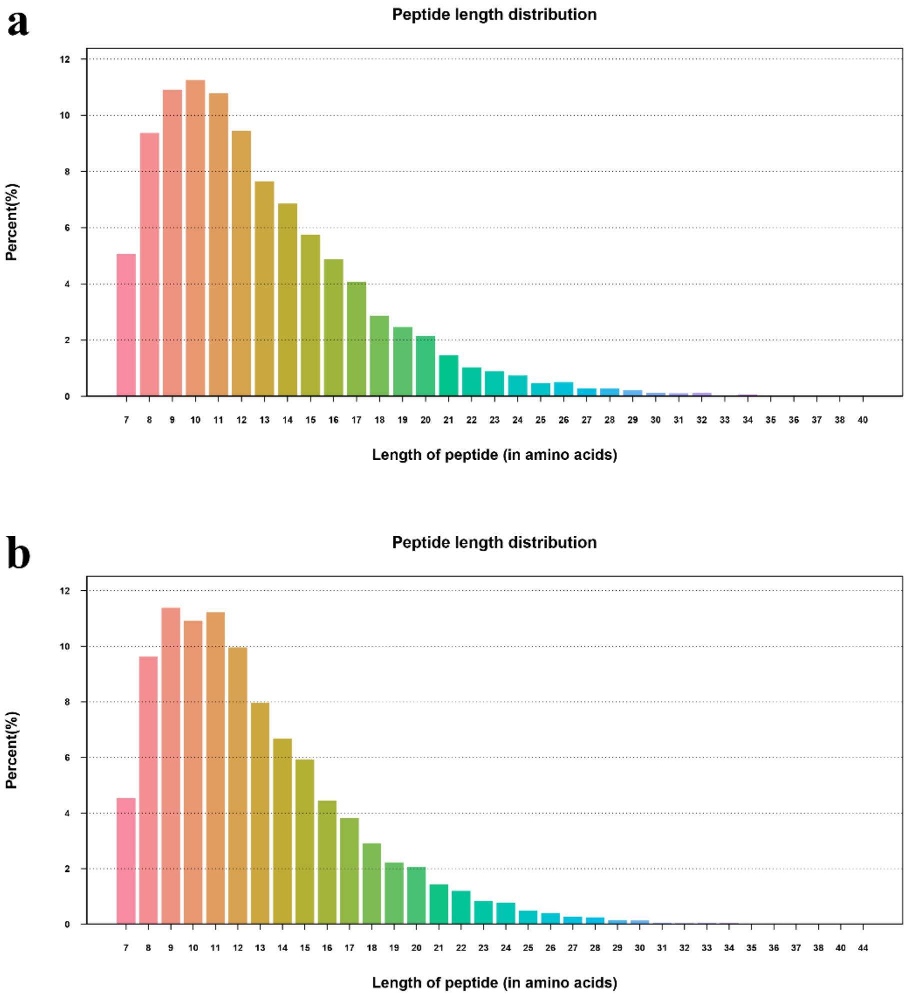


**Figure S1. Distribution of peptide length in leaf (a) and root (b) of *B. vulgaris*.** The *x* axis represents the length of the peptides, and the *y* axis represents the number of proteins.

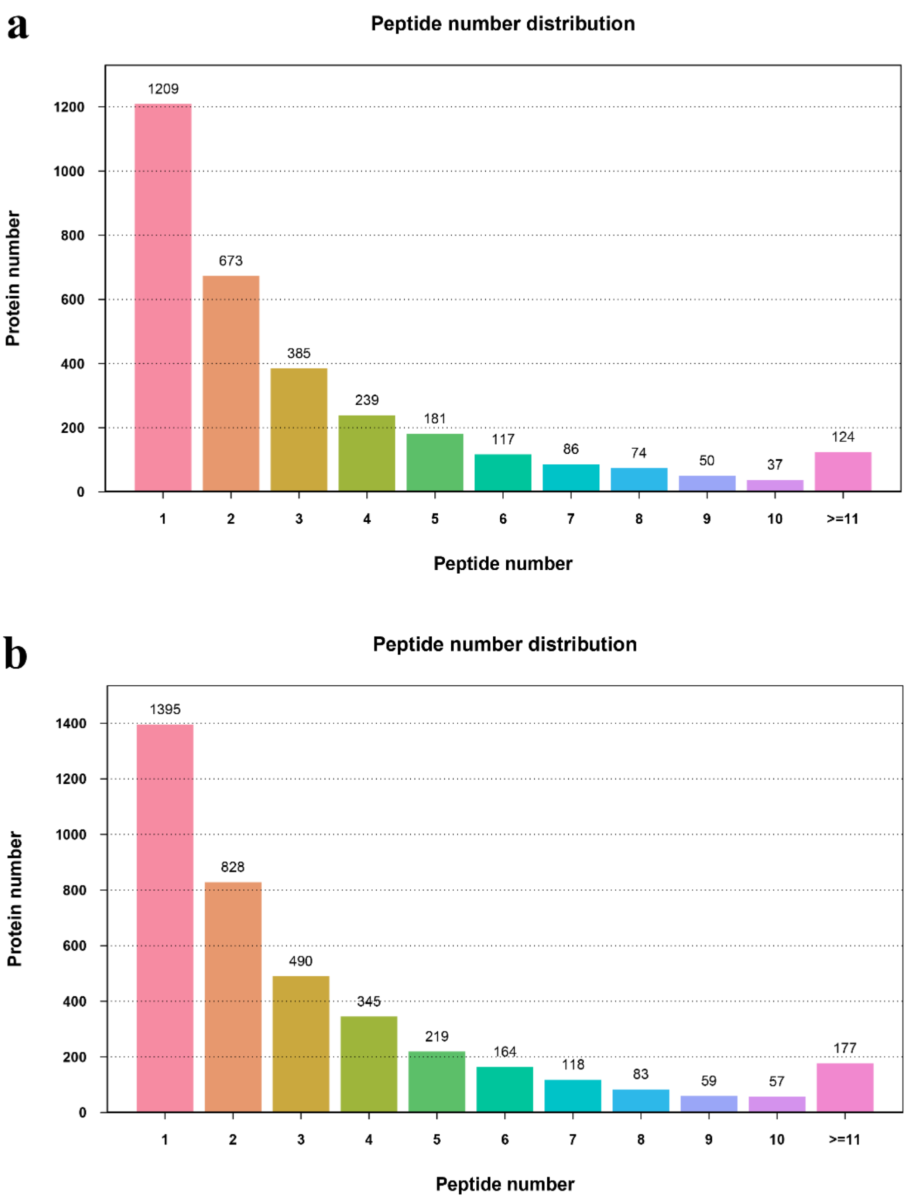


**Figure S2. Distribution of peptide number in leaf (a) and root (b) of *B. vulgaris*.** The *x* axis represents the scope of the number of identified peptides, and the *y* axis represents the number of proteins.

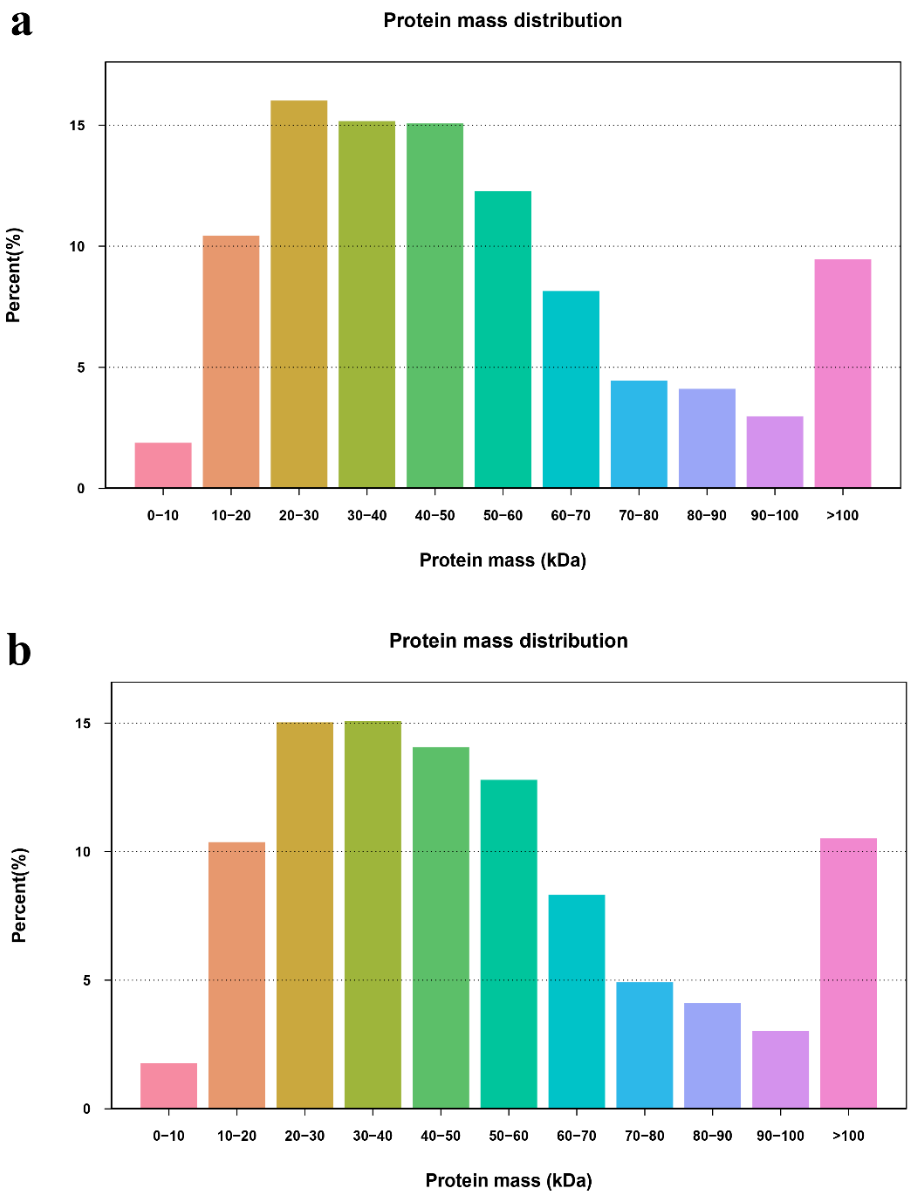


**Figure S3. Distribution of protein mass in leaf (a) and root (b) of *B. vulgaris*.** The *x* axis represents the molecular weights (kDa) of the identified proteins, and the *y* axis represents the number of proteins.

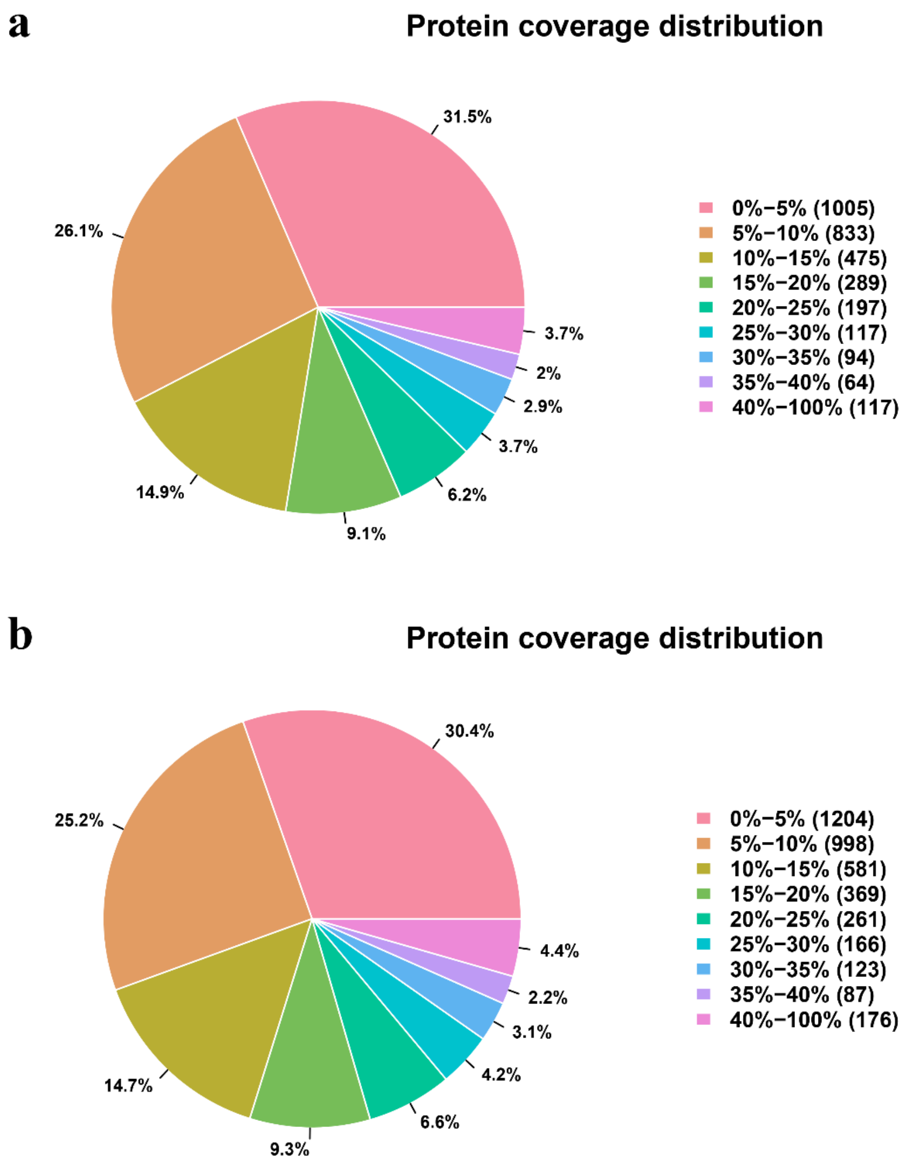


**Figure S4. Distribution of protein coverage in leaf (a) and root (b) of *B. vulgaris*.** The pie chart displays the proportion of the number of the different proteins within the scope of coverage in the total protein amount.
